# Combined dehydrogenation of glycerol with catalytic transfer hydrogenation of H_2_ acceptors to chemicals: Opportunities and challenges

**DOI:** 10.3389/fchem.2022.962579

**Published:** 2022-08-22

**Authors:** Guangyu Zhang, Jian Zhao, Xin Jin, Yanan Qian, Mingchuan Zhou, Xuewu Jia, Feng Sun, Jie Jiang, Wei Xu, Bing Sun

**Affiliations:** ^1^ State Key Laboratory of Safety and Control for Chemicals, SINOPEC Research Institute of Safety Engineering Co., Ltd., Qingdao, Shandong, China; ^2^ State Key Laboratory of Heavy Oil Processing, College of Chemical Engineering, China University of Petroleum, Qingdao, Shandong, China

**Keywords:** glycerol, lactic acid, H_2_ acceptor, CO_2_, dehydrogenation, catalytic transfer hydrogenation

## Abstract

Catalytic transformation of low-cost glycerol to value-added lactic acid (LA) is considered as one of the most promising technologies for the upgradation of glycerol into renewable products. Currently, research studies reveal that anaerobic transformation of glycerol to LA could also obtain green H_2_ with the same yield of LA. However, the combined value-added utilization of released H_2_ with high selectivity of LA during glycerol conversion under mild conditions still remains a grand challenge. In this perspective, for the first time, we conducted a comprehensive and critical discussion on current strategies for combined one-pot/tandem dehydrogenation of glycerol to LA with catalytic transfer hydrogenation of H_2_ acceptors (such as CO_2_) to other chemicals. The aim of this overview was to provide a general guidance on the atomic economic reaction pathway for upgrading low-cost glycerol and CO_2_ to LA as well as other chemicals.

## Introduction

### Background

**FIGURE 1:**
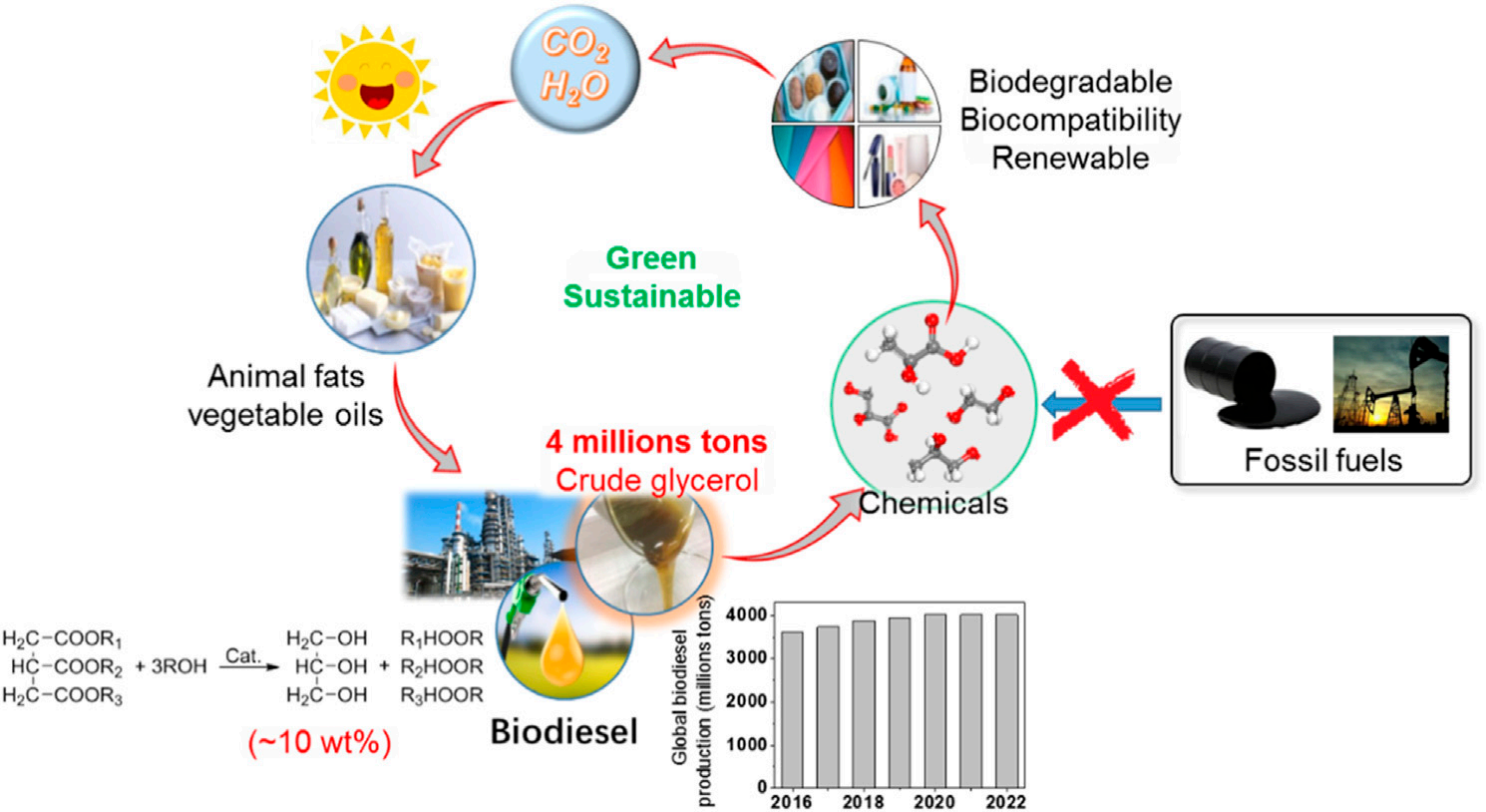
Global biodiesel production change in 2016–2022 years (millions liters).

Rapid consumption of fossil-based energy and materials has released major pollutants such as carbon dioxide, nitrogen oxides, and sulfur oxides, leading to significant environmental issues such as air pollution and global warming in our society. To address this challenge, renewable fuels and chemicals from catalytic conversion of biomass-derived feedstocks have gained increasing attention in the past decades. Among various renewable energies, biodiesel is considered as a good candidate for petroleum diesel due to its biodegradability, higher cetane number and engine lubricity, clean and environmental friendly nature. Biodiesel composed of mono-alkyl esters of long-chain fatty acids is derived from vegetable oils, animal fat, microalgae, and even waste cooking oils by the triglyceride-methanol or ethanolysis transesterification reaction (Abidin et al., 2012; Aboelazayem et al., 2018; Aboelazayem et al., 2019). In general, the production of biodiesel also yields a large amount of glycerol, about 10 wt% of the total biodiesel production. Rapid growth in the biodiesel industry due to its cleanness, high efficiency, and sustainability has resulted in excessive glycerol (about 4,000,000 tons per year, Figure 1), leading to a sharp drop in glycerol price (Ayoub and Abdullah, 2012; Nguyen et al., 2018). Furthermore, the current cost of biodiesel is still not competitive with diesel fuel. Therefore, economical optimization of biodiesel industry also motivated us to upgrade glycerol to valuable chemicals. In general, as a chemical building block, the glycerol can be converted to a series of value-added chemicals, such as lactic acid (LA), propanediol (PDO), ethylene glycol (EG), glyceric acid, dihydroxyacetone, glycolic acid, and tartronic acid. (Zhang et al., 2019; Meng et al., 2020; Liu et al., 2021; Sun et al., 2021; Yan et al., 2021; Zhang et al., 2021; Md Rahim et al., 2022). These products are widely used in food, medicine, organic synthesis, chemical industry, and other fields.

**FIGURE 2:**
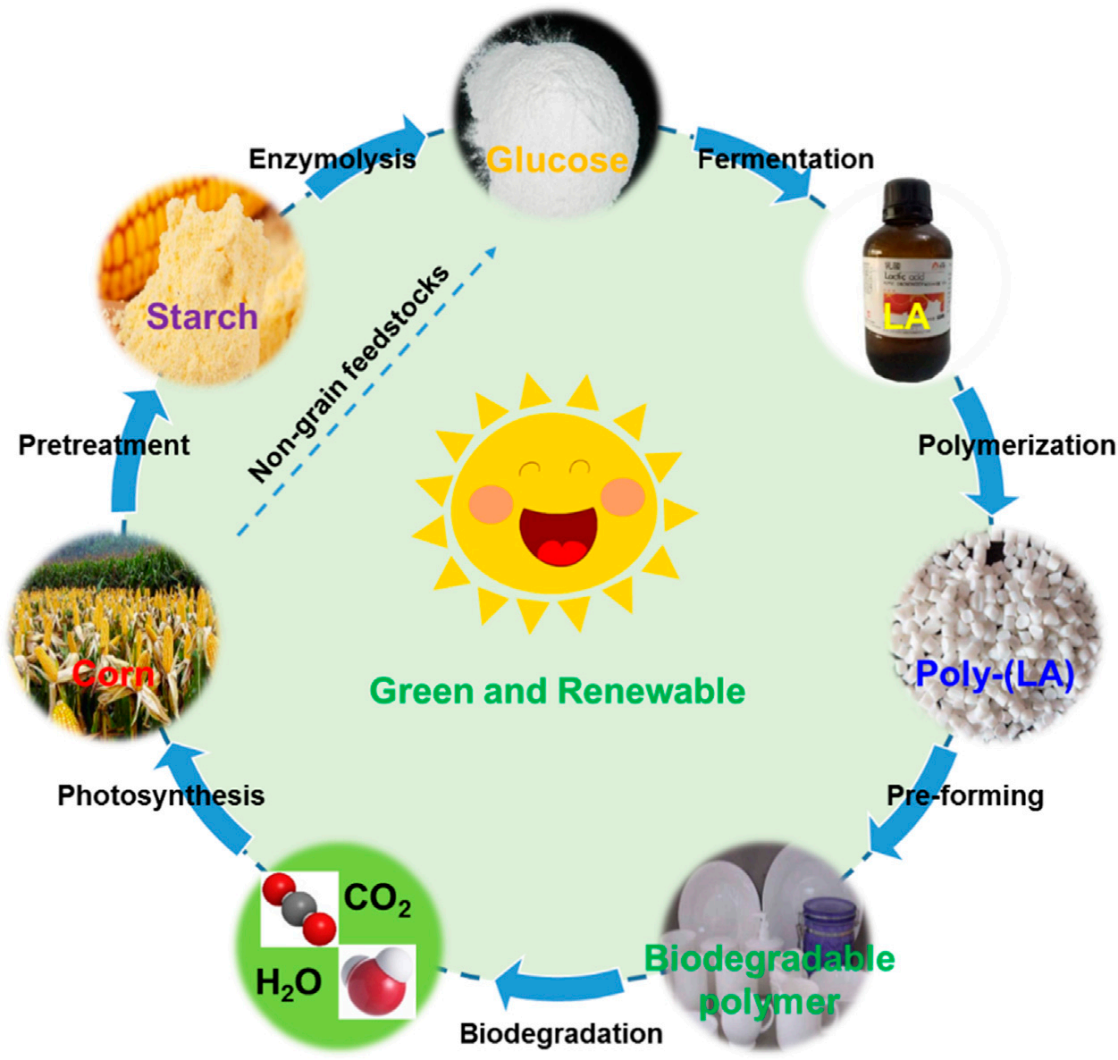
Diagram of nature circulation of poly-(LA).

Lactic acid, a α-hydroxyl carboxylic acid, is considered as an important bio-based platform chemical with great application prospects (nWim Groot et al., 2010). It has been widely used in many fields, such as food, cosmetic, leather, pharmaceutical, and textile industries. It is important to highlight that LA can be applied as a monomer to synthesize biodegradable poly-(LA). Due to its biocompatibility and biodegradability, poly-(LA) is considered as a potential candidate for conventional petroleum-based polymers, such as polyethylene terephthalate, polystyrene, and polypropylene (Jamshidian et al., 2010; Djukić-Vuković et al., 2019; Luo et al., 2020). Non-degradable plastic has become a significant environmental issue on cultivated land and marine organism. To address this challenge, poly-(LA), a sustainable biodegradable polymer, has gained increasing attention in recent years (Figure 2). The annual production of poly-(LA) is estimated to be 830,000 tons in 2020 (Dreschke et al., 2015), which means a high demand for lactic acid monomers in the future. The demand for LA exceeds the supply, which drives us to increase the production efficiency of LA.

### Production of LA

**FIGURE 3:**
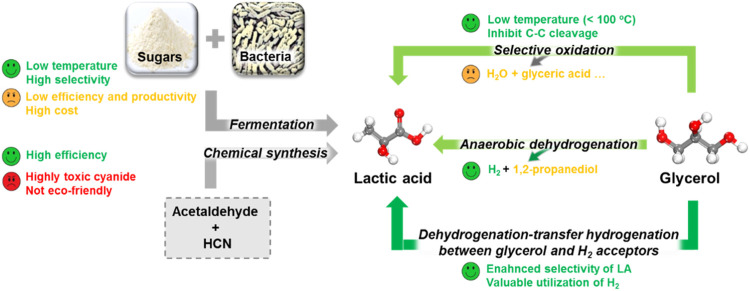
Comparison of various LA production processes (Zhang et al., 2019).

Up to date, conventional bio-fermentation of readily available sugars with microorganisms is still the major LA source, which displays advantages of utilization of renewable substrates, low processing temperature, low energy consumption, and production of optically pure D- or L-LA in the appropriate bacteria (Budhavaram and Fan, 2009; Nguyen et al., 2012; Abdel-Rahman et al., 2013; Tang et al., 2016; Djukić-Vuković et al., 2019). However, several bottlenecks limited its development to satisfy the fast-growing LA market (Figure 3) (Wang et al., 2015; Zhang et al., 2019). One limiting factor is the high cost because hydrolyzing renewable materials to remove their lignin is difficult in pretreatment processes. In addition, the difficult purification of complex fermentation productions also hampers downstream processes. Another bottleneck is very low efficiency and productivity of the fermentation method due to a long fermentation time, low concentration of substrates, and complex separation and purification. Therefore, the fermentation method may not meet the increasing market demand of LA in the future. In addition, another important method is the chemical synthesis of LA using acetaldehyde and HCN, showing a high productivity and efficiency. However, it is of less interest currently because of safety and environmental concerns (Shen et al., 2019). Hence, it is urgent to develop new technical routes for environmental friendly, cost-effective, and large-scale production of LA from abundant biomass with less energy and capital intensity.

**SCHEME 1:**
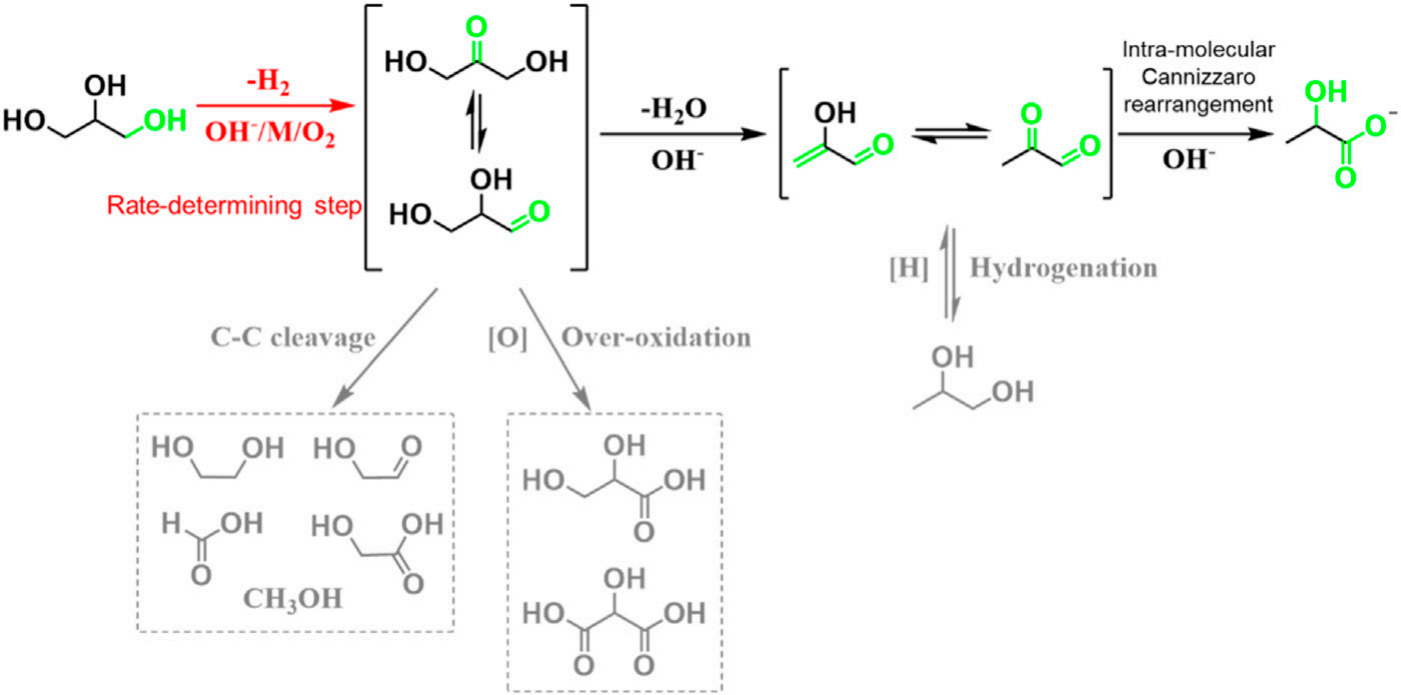
Reaction pathways for the conversion of glycerol to LA.

In the past few decades, both experimental and theoretical studies have demonstrated that biomass and derived carbohydrates, including cellulose, glucose, fructose, hexose, and glycerol can be transformed into high-valued LA and other chemicals (Dusselier et al., 2013; Maki-Arvela et al., 2014; Razali and Abdullah, 2017; Lari et al., 2018; Li et al., 2018; Nda-Umar et al., 2018; Zavrazhnov et al., 2018; Kim and Moon, 2019; Li et al., 2019; Maki-Arvela et al., 2020). Among these biomass feedstocks, glycerol, as a byproduct of biodiesel production, has attracted most attention in the catalytic transformation of biomass to LA. Catalytic conversion of glycerol to LA is a promising candidate route to replace the bio-fermentation technique due to the advantages of its green nature, high efficiency, and productivity as well as cost-effectiveness, which can both upgrade the cheap glycerol and meet the growing demand for the LA market. Many reaction systems including aerobic and anaerobic have been developed in the past decades for the conversion of glycerol to LA. Both experimental and theoretical studies have confirmed that three main steps are involved in the catalytic conversion of glycerol into LA (Scheme 1), including 1) the C–H and O–H bond cleavage to glyceraldehyde or dihydroxyacetone and H_2_O or H_2_, 2) the C–O bond cleavage to pyruvaldehyde, and 3) intramolecular Cannizzaro rearrangement of pyruvaldehyde to LA (Jin et al., 2013; Zhang et al., 2019). It is generally known that the activation of C–H bond is regarded as the key reaction step in these cascade reactions (Li et al., 2019; Zhang et al., 2019). To improve the activity of C–H bond cleavage, many reaction systems, including aerobic and anaerobic, and a series of homogenous and heterogeneous catalysts have been developed in the past decades (Razali and Abdullah, 2017; Zavrazhnov et al., 2018; Li et al., 2019).

Recent reviews have detailed and summarized various catalyst types, compositions, performances, stability, and reaction parameters including the base promoter and gas atmosphere, as well as their reaction networks. For example, Razali and Abdullah (2017) provided an extensive overview on the production of LA from glycerol by elucidating the roles of metal particle sizes and distribution, base promoters, metal and support as well as reaction atmosphere. In the last year, Wang et al. summarized alkali-promoted and alkali-free catalytic systems in detail, and discussed the effect of H_2_ (released from dehydrogenation of glycerol) on product distribution (Li et al., 2019). However, there is lack of systematic summary on the atomic economic design of the reaction system with regard to H_2_ released from dehydrogenation of glycerol. To our best knowledge, anaerobic transformation of glycerol to LA could also obtain H_2_ with the same yield of LA at the same time, while the released hydrogen finally generated worthless H_2_O under O_2_ pressure, which is an atomic uneconomic reaction pathway. The released hydrogen in the hydrogenation reaction could participate in converting glycerol to value-added propanediol and ethylene glycol, because the metallic catalysts are active for both dehydrogenation and hydrogenation, which is not desirable due to the original intention of producing LA. Several research studies have demonstrated that adding hydrogen acceptor to the reaction system is feasible for preventing the hydrogenation reaction of intermediate such as pyruvaldehyde with *in situ* generated H_2_. Recent years have witnessed the development of combined dehydrogenation and catalytic transfer hydrogenation between glycerol and H_2_ acceptors. Therefore, in this review, we will focus on combined dehydrogenation of glycerol with catalytic transfer hydrogenation of H_2_ acceptors to value-added chemicals.

## Catalytic conversion of glycerol to LA

### Aerobic reaction and mechanism

Selective oxidation of glycerol has been demonstrated to be thermodynamically more favorable for C–H bond activation under mild reaction conditions (e.g., lower operating temperature and alkali concentration), which greatly reduces energy consumption (Shen et al., 2010; Lakshmanan et al., 2013; Tao et al., 2017; Evans et al., 2020; Tao et al., 2020; Torres et al., 2021). Furthermore, low reaction temperature can also significantly inhibit C–C bond cleavage by alkalis, thus good selectivity of C_3_ products. Selective oxidation of glycerol to LA is generally carried out with the promotion of various noble metal and some non-noble metal catalysts (e.g., Au, Pt, Pd, and polyoxometalate ) (Shen et al., 2010; Lakshmanan et al., 2013; Xu et al., 2013; Cho et al., 2014; Purushothaman et al., 2014; Zhang et al., 2016a; Zhang et al., 2016b; Arcanjo et al., 2017; Tao et al., 2017; Zhang et al., 2017; Douthwaite et al., 2020; Evans et al., 2020; Tao et al., 2020; Torres et al., 2021; Wang et al., 2021). In the first important advances, Shen et al. (2010) reported that the bimetallic Au–Pt catalysts exhibit excellent performances with a high yield of 86% in the presence of alkali and O_2_ at 90°C. Much lower reaction temperature significantly limits the C–C bond cleavage, leading to favorable lower selectivity of C_2_ and C_1_ products. However, some of the glyceric acid as the main byproduct was formed due to the over-oxidation reaction. Mechanism studies reveal that oxidative dehydrogenation of glycerol to intermediates, including dihydroxyacetone and glyceraldehyde, is the key step during selective oxidation of glycerol to LA (Li et al., 2019; Zhang et al., 2019). Strong interaction and synergism effect in Au and Pt play a great role in promoting oxidative dehydrogenation of glycerol to dihydroxyacetone and glyceraldehyde. After that, the resulting intermediates undergo dehydration and subsequently benzylic acid rearrangement (some research studies proposed an internal Cannizzaro reaction) (Yin et al., 2016; Yin et al., 2017; Li et al., 2019) to LA in the presence of NaOH. As the main by-product, glyceric acid could be generated by further oxidation of glyceraldehyde over bimetallic Au–Pt catalyst under high O_2_ pressure. In addition, the deep-oxidation products, including tatronic acid, glycolic acid, oxalic acid, and formic acid (FA), could also be inevitably formed in the presence of metal catalysts and O_2_, which reduce the selectivity of LA (Wang et al., 2013; Douthwaite et al., 2020; Yan et al., 2020).

**SCHEME 2:**
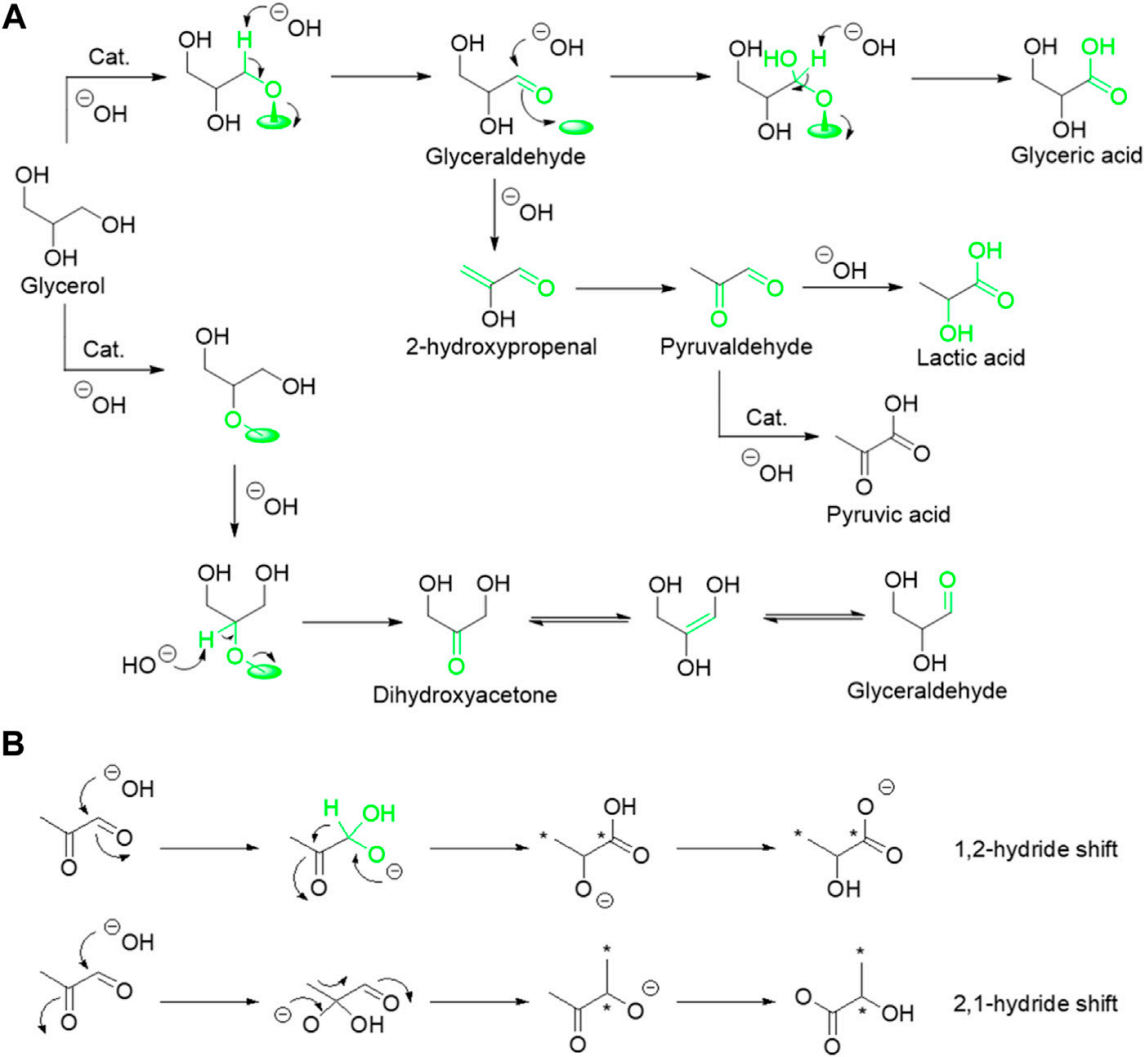
Mechanisms under alkaline conditions: **(A)** transformation of glycerol; **(B)** transformation of pyruvaldehyde.

Despite fast progress in this research field, the mechanism for the formation of LA is still a subject of contention, especially the competitive pathway in dehydration of glyceraldehyde and C–C bond cleavage as well as the nature of rearrangement reaction. Recently, Evans et al. (2020) thoroughly studied the formation mechanism of LA from glycerol by conducting a series of isotopic labeling experiments with 1.3–^13^C glycerol using a model AuPt/TiO_2_ catalyst. The reaction conditions, including reaction temperature, pH, and O_2_ pressure, are highly influential on both the conversion rate of glycerol and product distribution (Scheme 2A). They found that catalyst, high reaction temperature, and high O_2_ pressure are favorable for oxidative dehydrogenation of glycerol to mixture intermediate products of dihydroxyacetone and glyceraldehyde, while pH is independent for this rate-determining step. Then, the resulting dihydroxyacetone and glyceraldehyde could undergo dehydration to 2-hydroxypropenal and isomer of pyruvaldehyde. Meanwhile, an additional competitive reaction pathway of sequential oxidation of glyceraldehyde and C–C bond cleavage occurs under O_2_ pressure. Notably, selectivity of LA can be significantly enhanced with the increase of the base content in the reaction system, indicating that the dehydration of glyceraldehyde to pyruvaldehyde is favored over its sequential oxidation and C–C bond cleavage reaction (Purushothaman et al., 2014). Hence, high yield of LA over glyceric acid was achieved during selective oxidation of glycerol to LA. Furthermore, isotopic labeling experiments with 1.3–^13^C glycerol are conducted to elucidate the formation mechanism of LA from the intermediate of pyruvaldehyde (Scheme 2B). They found that ^13^C signals could be detected in both the carboxylic acid and methyl groups in LA with similar quantities, suggesting that the formation of LA from pyruvaldehyde undergo a base-catalyzed 1,2-hydride shift (intramolecular Cannizzaro reaction) rather than 2,1-methide shift (benzylic acid rearrangement).

### Anaerobic reaction and mechanism

Different from selective oxidation strategy, anaerobic transformation of glycerol to LA can avoid the over-oxidation reaction, and release H_2_ (in almost the same mole yield as LA) rather than a worthless H_2_O molecule. Thus, it could provide a higher LA yield and atomic economy, which is consistent with (Razali and Abdullah, 2017) the evolution of the modern chemical industry. It is known that H_2_ is an important chemical raw material, widely used in the ammonia synthesis, petrochemical, Fishcher–Tropsch process, and clean energy industry. Hence, several strategies have been developed to value-added utilization of hydrogen produced from C–H and O–H bond cleavage of glycerol (Cortright et al., 2002; Davda et al., 2005; Wen et al., 2008). Currently, various homogeneous or solid metal catalysts have been developed to catalyze glycerol dehydrogenation to LA, and release H_2_ at the same time (Tang et al., 2019a; Zhang et al., 2019; Ainembabazi et al., 2020; Bharath et al., 2020; Feng et al., 2020; Heltzel et al., 2020; Valekar et al., 2021; Zhang et al., 2021). For example, in alkali-catalyzed hydrothermal conversion systems, the C–H and O–H groups of glycerol can undergo a nucleophilic attack by OH^−^ to form intermediates of glyceraldehyde or dihydroxyacetone. The intermediates subsequently undergo C–O bond cleavage and rearrangement affording lactate (Hisanori et al., 2005; Shen et al., 2009; Xu et al., 20112011). Alkali-catalyzed conversion of glycerol to LA can be carried out in several hours, addressing the low efficiency and low productivity of bio-fermentation method. A typical example, Hisanori et al. (2005) reported that hydrothermal transformation of glycerol catalyzed by NaOH showed a LA yield of 90% in 1.5 h at 300°C. However, harsh reaction conditions, such as high reaction temperature (e.g., 300°C) and high concentration of alkali (e.g., 4 mol/L), are generally needed, because the C–H bond activation is an energy-demanding process. In addition, under the harsh reaction conditions, C–C bond cleavage is favorable, leading to the formation of side products reducing selectivity of LA.

Currently, a series of homogeneous or solid metal catalysts, including Ir- (Sharninghausen et al., 2014; Lu et al., 2016; Finn et al., 2018), Pt- (Jin et al., 2013; Ftouni et al., 2015; Oberhauser et al., 2016; Tang et al., 2019b; Zhang et al., 2019), Pd- (Marques et al., 2015; Shen et al., 2019), Ru (Deng et al., 2021), Au- (Shen et al., 2017a; Palacio et al., 2019), Cu- (Roy et al., 2011; Moreira et al., 2016; Yang et al., 2016; Yin et al., 2016; Shen et al., 2017b; Li et al., 2017; Yin et al., 2017; Palacio et al., 2018a), Ni- (Qiu et al., 2018; Yin et al., 2018; Abdullah et al., 2020; Tang et al., 2020; Xiu et al., 2020), and Co-based (Palacio et al., 2018b) systems, have been developed to promote the rate-determining step under relatively mild reaction conditions (lower reaction temperature and alkali concentration). For example, our previous report (Zhang et al., 2019) indicates that Pt–Co bimetallic catalysts significantly enhance the rate of C–H and O–H bond cleavage, showing a good dehydrogenation activation for glycerol transformation at 200°C (glycerol conversion: 85%, LA selectivity: 88%). Same as aerobic transformation of glycerol, the alkalis or other solid acid/base sites exhibit a strong promotion effect for sequential dehydration and intramolecular Cannizzaro reaction. However, at such high reaction temperature, the base could catalyze retro-aldolization reaction of glyceraldehyde, leading to C–C bond cleavage, which reduces the final yield of LA. Based on the detailed studies of reaction pathways in previous works (Jin et al., 2013; Yfanti and Lemonidou, 2018), it is clear that the released hydrogen in the hydrogenation reaction forms value-added propanediol and ethylene glycol, because the metallic catalysts are active for both dehydrogenation and hydrogenation, thus showing good atomic efficiency. However, in previous works, the formation of by-products, including propanediol, ethylene glycol, and deep reduction products such as various alkanes, significantly reduces the LA selectivity, which is not desirable due to the original intention of producing LA.

## One-pot dehydrogenation and catalytic transfer hydrogenation between glycerol and H_2_ acceptor

**FIGURE 4:**
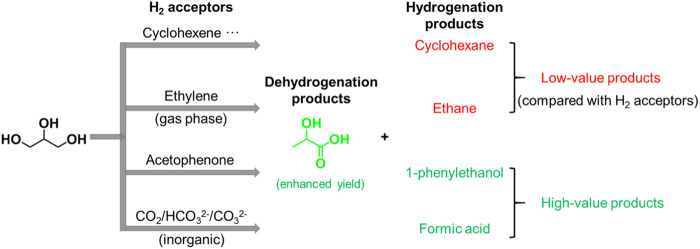
Dehydrogenation and catalytic transfer hydrogenation of glycerol and H_2_ acceptor.

To improve the LA yield, the hydrogen produced by C–H and O–H cleavage need to be consumed in time. Several research studies have demonstrated that adding hydrogen acceptor to the reaction system is feasible for preventing the hydrogenation reaction between intermediate such as pyruvaldehyde with released H_2_ from glycerol dehydrogenation (Figure 4) (Sharninghausen et al., 2015; Oberhauser et al., 2016; Tang et al., 2019b; Ainembabazi et al., 2020; Heltzel et al., 2020; Tang et al., 2020; Deng et al., 2021; Valekar et al., 2021). We will give a detailed overview about one-pot dehydrogenation and catalytic transfer hydrogenation between glycerol and H_2_ acceptors.

### Unsaturated hydrocarbon and carbonyl chemicals as H_2_ acceptors

**SCHEME 3:**
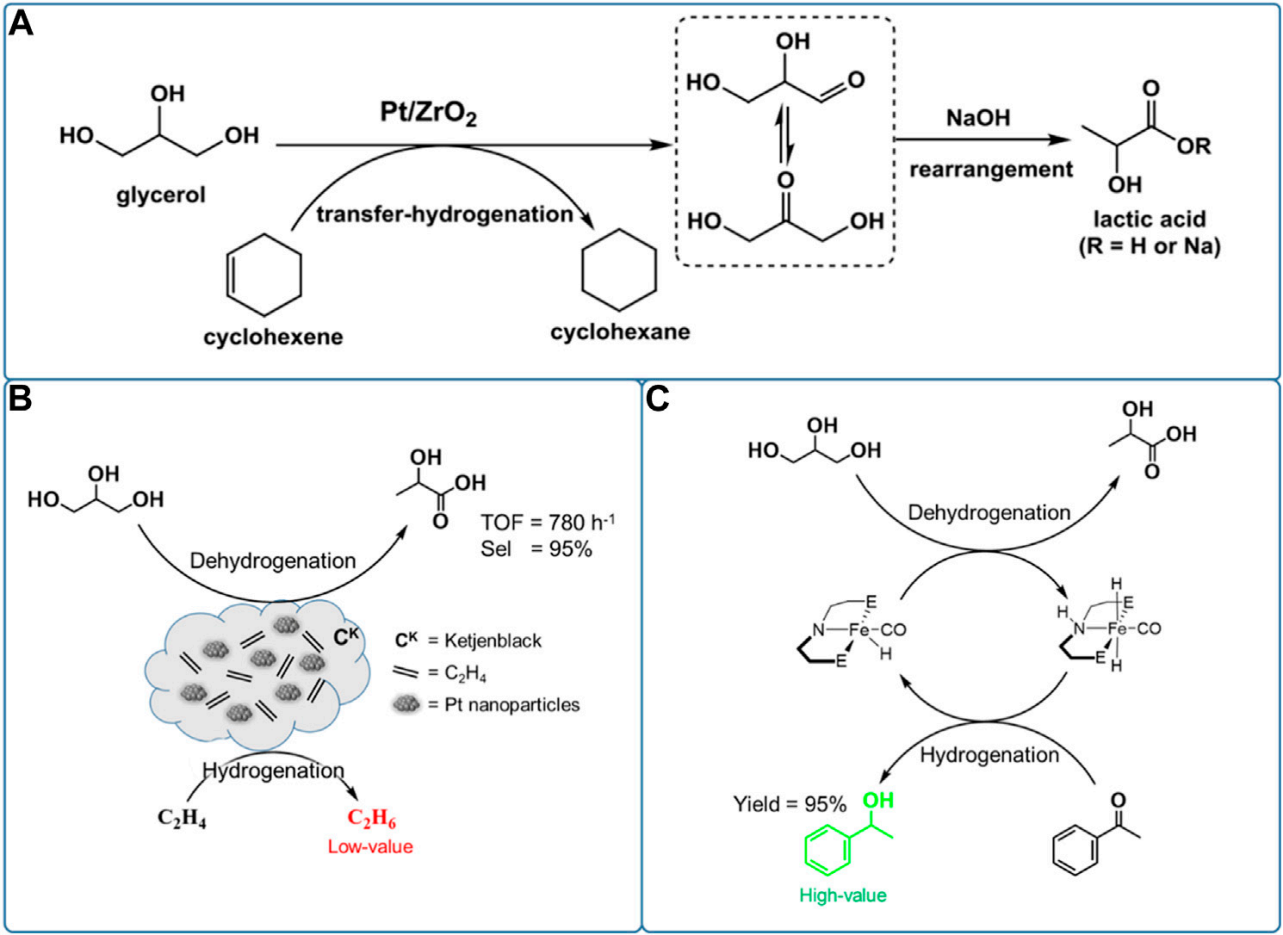
Catalytic reaction routes from glycerol to lactic acid with various H2 acceptors: **(A)** cyclohexene, **(B)** high pressure ethylene, and **(C)** acetophenone.

In the first important advances, Tang et al. (2019b) reported that adding an organic phase of cyclohexene to glycerol aqueous solution can consume the released H_2_ from glycerol dehydrogenation, preventing undesired hydrogenation reaction (Scheme 3A). They synthesized a series of highly dispersed Pt-based catalysts (atomically dispersed Pt species, sub-nanometer Pt clusters, and extra-fine Pt nanoparticles) supported by nanosized ZrO_2_ *via* optimization of the loading of Pt and calcination as well as reduction temperature. The high dispersed 2Pt/ZrO_2_-550-R250 catalysts with a narrow size distribution centered at 1.4 nm and a relatively large loading (2 wt%) of Pt nanoparticles showed an unsurpassed 95% yield of LA at 96% conversion of glycerol at 160°C in 4.5 h under 20 bar N_2_ pressure. This is the highest LA selectivity (∼99%) in the previous works. The novel catalytic system also leads to a selectivity of 36% in catalytic transfer hydrogenation from glycerol to cyclohexene. Apart from cyclohexene, 1-decene was also used as an H_2_ acceptor achieving similarly remarkable LA selectivity of 99% at glycerol conversion of 97%, while giving a significantly higher selectivity in catalytic transfer hydrogenation (92%). However, a partial deactivation of the Pt-based catalyst occurs following the aggregation of high dispersed Pt nanoparticles into larger ones (ca. 5 nm).

In order to find a significantly cheaper alternative to precious Pt-based catalysts, they investigated a series of Ni-based bimetallic catalysts for conversion of glycerol to LA, which have both good dehydrogenation and hydrogenation capacities (Tang et al., 2020). The bimetallic NiCo catalyst supported on CeO_2_ gave a much higher catalytic activity than the monometallic Ni/CeO_2_ or Co/CeO_2_ catalysts, during the conversion of glycerol to LA with concomitant transfer hydrogenation of various H_2_ acceptors (including cyclohexene, 1-decene, levulinic acid, nitrobenzene, and benzene). Combining characterization and reaction data proved that the Ni species are major active sites, but the incorporation of Co could promote dispersion and stability of Ni species on CeO_2_, thus leading to a remarkable LA yield of 93% at glycerol conversion of 97% at 160°C and 6.5 h under 20 bar N_2_ pressure. Furthermore, compared with other cheap metal catalysts, the bimetallic NiCo/CeO_2_ catalyst showed a remarkable catalytic performance in dehydrogenation of glycerol to LA under relatively milder reaction conditions. In addition, the recycle study revealed that the NiCo/CeO_2_ catalyst showed a good reusability, no loss of the original activity after three runs.

To enhance the formation of LA, supplying ethylene gas rather than liquid phase H_2_ acceptor to the one-pot dehydrogenation and catalytic transfer hydrogenation systems has also been demonstrated to be feasible during conversion of glycerol (Scheme 3B). Recently, Oberhauser et al. (2016) synthesized a series of Pt-based nanoparticle catalysts supported on Ketjenblack (C^K^) with a high surface area (∼1,400 m^2^/g), *via* the metal vapor synthesis method, used in conversion of glycerol to LA. The Pt@C^K^ with small-sized Pt nanoparticles (mean size of 1.5 nm) showed a high LA selectivity of 95% at a glycerol conversion of near 100% at 140°C and 6 h under 875 psi ethylene pressure. Adding ethylene gas to the reaction system not only consumes the released H_2_ from glycerol dehydrogenation, preventing the undesired hydrogenation reaction, but also improves the conversion of glycerol. In the absence of ethylene, the Pt@C^K^ catalyst showed poor catalytic performances with a low glycerol conversion (44%) and LA selectivity (64%), but high 1,2-PDO selectivity (36%) at 140°C and 3 h. However, with ethylene gas as an H_2_ acceptor (875 psi), the Pt@C^K^ catalyst showed a significantly enhanced LA selectivity (95%) and 1,2-PDO was not observed at an increased glycerol conversion of 59%. Combining characterization and reaction data proved that the ethylene gas can stabilize together with high dispersed Pt nanoparticles (∼1.5 nm) through reversible metal atom coordination, inhibiting sintering of Pt nanoparticles. In addition, the recycle study revealed that the Pt@C^K^ catalyst showed a good reusability, no loss of the original activity after three runs.

As mentioned earlier, various H_2_ acceptors, especially ethylene and cyclohexene, significantly enhance the formation of LA during one-pot tandem dehydrogenation and catalytic transfer hydrogenation of glycerol, which is greatly consistent with our original intention for producing LA from dehydrogenation of glycerol. However, the ethylene and cyclohexene were transformed into the cheaper alkane, which is undesirable. To obtain the more valuable hydrogenation products, several other unsaturated compounds have been selected as H_2_ acceptors replacing undesirable olefin (Scheme 3C). In the first important advances, Sharninghausen et al. (2015) synthesized a series of iron complexes of PNP pincer ligands for homogeneous conversion of glycerol at 140 °C and 6 h, leading to LA selectivity of 88% at glycerol conversion of 39%. Meanwhile, several studies have demonstrated that the Fe-PNP complex catalysts showed good activity for the hydrogenation of alcohols, esters, and N-heterocycles (Chakraborty et al., 2014a; Chakraborty et al., 2014b; Qu et al., 2014). Given the hydrogenation capacity of the Fe-PNP complexes for several unsaturated compounds, they studied combined dehydrogenation and catalytic transfer hydrogenation between glycerol and acetophenone. Surprisingly, the acetophenone was hydrogenated to 1-phenylethanol with a high yield of 95% at 120°C for 22 h. Notably, the hydrogenation product of 1-phenylethanol is an upgraded chemical than acetophenone, which is favorable in the economic area. However, the reaction performances of glycerol in this system are not analyzed in more detail.

### CO_2_ and its derivatives as H_2_ acceptors

**SCHEME 4:**
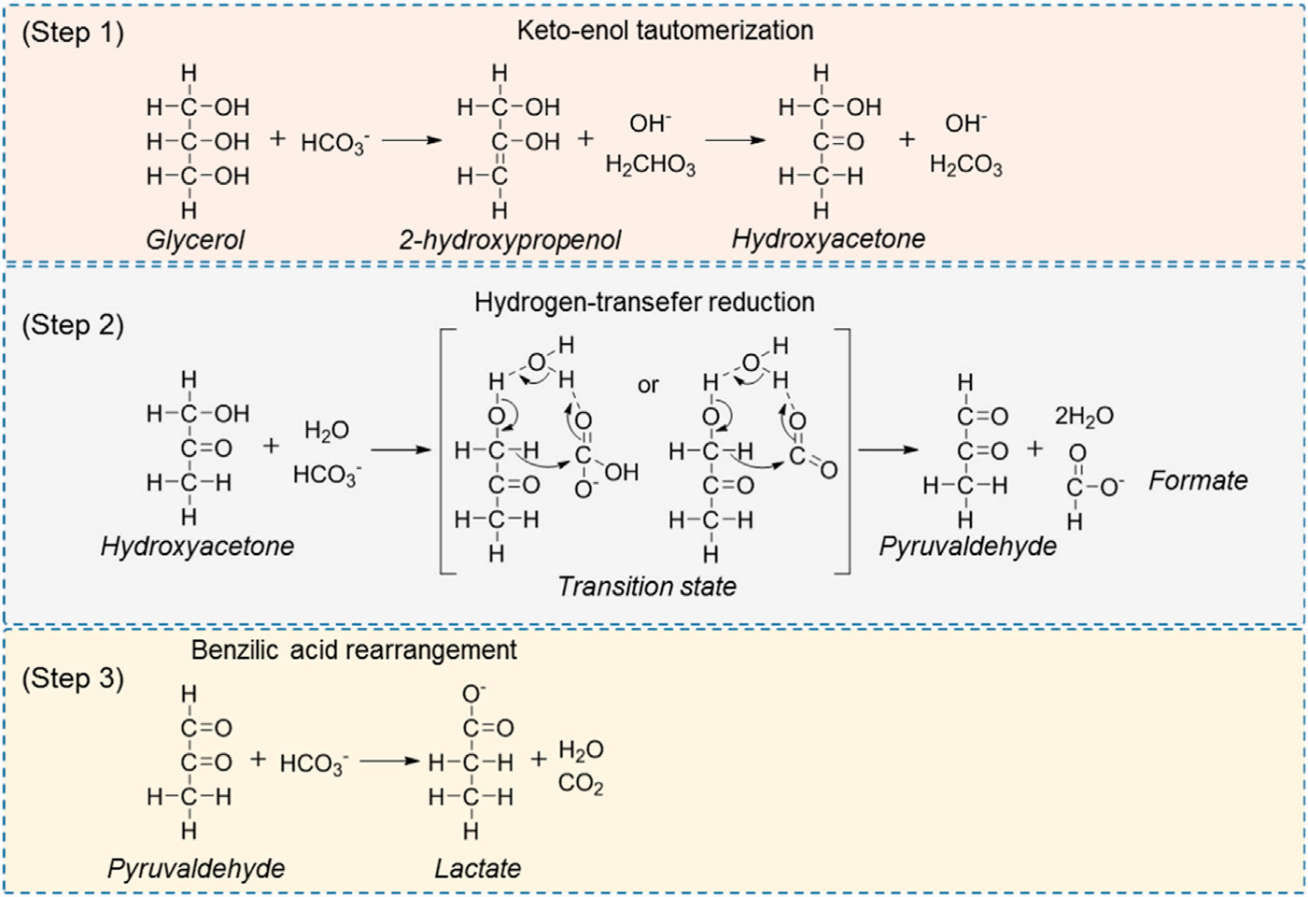
Proposed pathway of the hydrogen-transfer reduction of NaHCO_3_ with glycerol (Shen et al., 2012).

One-pot dehydrogenation and catalytic transfer hydrogenation of glycerol and CO_2_/carbonate/bicarbonate to afford LA and formic acid (FA) is another attractive path to upgrading both low-value feedstocks, given the abundance of glycerol and CO_2_ as renewable materials (Kovács et al., 2006; Shen et al., 2012; Shen et al., 2014; Su et al., 2014; Wang et al., 2016; Heltzel et al., 2018). In the first important advances, Jin et al. (Shen et al., 2012; Shen et al., 2014; Wang et al., 2016) reported a non-catalyzed transfer hydrogenation of CO_2_/NaHCO_3_ with glycerol under alkaline hydrothermal conditions to co-production of LA and FA. In this process, the glycerol was used as a reducing agent and converted to LA with a high yield of about 90%, while the NaHCO_3_ was converted to FA with a same excellent yield such as LA at 300°C in 1.5 h. The effects of various parameters, for example, CO_2_, D_2_O solvent effect, reactor materials effect, and H_2_O molecule catalysis were investigated in detail to disclose the possible reaction mechanism. Based on the experimental data and theoretical analysis, they proposed a plausible reaction pathway as shown in Scheme 4. They claimed that the glycerol is first converted to hydroxyacetone via a dehydration and keto-enol tautomerization reaction. Subsequently, the resulting hydroxyacetone, H_2_O and CO_2_ could form an eight-membered cyclic transition state *via* two hydrogen bonds. Following, an intramolecular hydride shift occurs in the cyclic transition state to form pyruvaldehyde and FA, accompanied by the release of a water molecule. Finally, the resulting pyruvaldehyde undergoes a benzylic acid rearrangement to form the LA. In the proposed pathway, the water molecules are connected with the substrate molecules via the hydrogen bond for the formation of the eight-membered ring network, which is the key step of the reaction for co-production of LA and FA from glycerol and CO_2_. In their works, one-pot hydrogen transfer of glycerol to CO_2_ for affording LA and FA has been demonstrated to be feasible. However, it is necessary to further optimize the reaction system to avoid the harsh reaction conditions (300°C).

Recently, Heltzel et al. (2018) compared the $\Delta {\text{G}}_{aq}^{o}$ of catalytic transfer hydrogenation and direct hydrogenation of CO_2_ in an aqueous solution (Table 1). When LA is the ultimate product from glycerol dehydrogenation, CO_2_ catalytic transfer hydrogenation shows a lower $\Delta {\text{G}}_{aq}^{o}$ of–9.21 kcal/mol, which is more favorable than direct hydrogenation from H_2_ ( $\Delta {\text{G}}_{aq}^{o}$ : 13.4 kcal/mol). In addition, they found that Ru N-heterocyclic carbine (NHC) complexes with sulfonate-functionalized wingtips are highly active for acceptor-less dehydrogenation of glycerol to LA (Heltzel et al., 2018). Hence, they tried to combine the dehydrogenation of glycerol and catalytic transfer hydrogenation of CO_2_ and bicarbonate to co-produce value-added LA and FA. Experimental data showed that this one-pot tandem hydrogen transfer reaction is a temperature-, base concentration-, and CO_2_ pressure-sensitive system. Equimolar amounts of LA and FA are formed (∼600 TON) at 150°C, while an increasing amount of LA than FA is formed at reaction temperature over 150°C. In addition, in the absence of KOH, no LA and FA are formed from a reaction at 150 °C. However, equivalent LA and FA (∼50 mM) are produced with a 330 TON at also 150°C, while the base concentration increased to 1 and 2 M. Notably, the reaction still affords ∼50 mM LA but greatly decreased FA, while the base concentration decreased to 0.25 M. Therefore, higher LA yield can be achieved with higher reaction temperature and lower base concentration. At 180°C, the Ru/NHC complexes show 1,685 and 1,065 TON of LA and FA in 24 h, respectively. The carbonate salts show a greatly enhanced TON for LA and FA of 42,610 and 3,588, respectively, because of good solubility than CO_2_ in the reaction system. Furthermore, they proposed a plausible reaction pathway. First, glycerol is adsorbed on Ru species, followed by deprotonation promoted by the base. Then, *β*-hydride elimination at the secondary position of glycerol occurs, forming Ru–H species and dihydroxyacetone (DHA). The intermediate of DHA is transformed to LA *via* tandem isomerization, dehydration, and the intramolecular rearrangement Cannizzaro reaction. The HCO_3_^−^ next binds to the Ru–H species and undergoes a hydroxide elimination (Kovács et al., 2006). The resulting H–Ru–CO_2_ transition state undergoes the insertion reaction to generate Ru-formate species, which further dissociates to formate.

**TABLE 1:** Calculated free energies of reaction ( $\Delta G_{\mathrm{aq}}^{o}$ ) for the CO_2_ direct hydrogenation and catalytic transfer hydrogenation (Gaussian16, G3B3, PCM water) (Heltzel et al., 2018).

Entry	$\Delta G_{\mathrm{aq}}^{o}$ (*kcal/mol*)
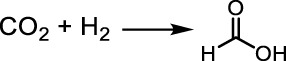	13.4
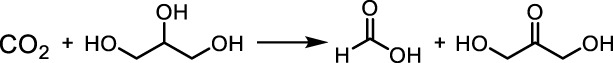	12.3
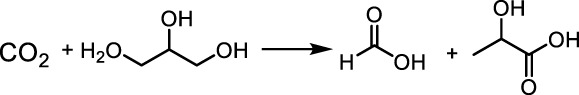	−9.2
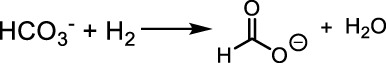	4.4
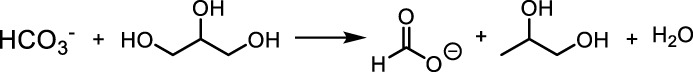	−83.9

**SCHEME 5:**
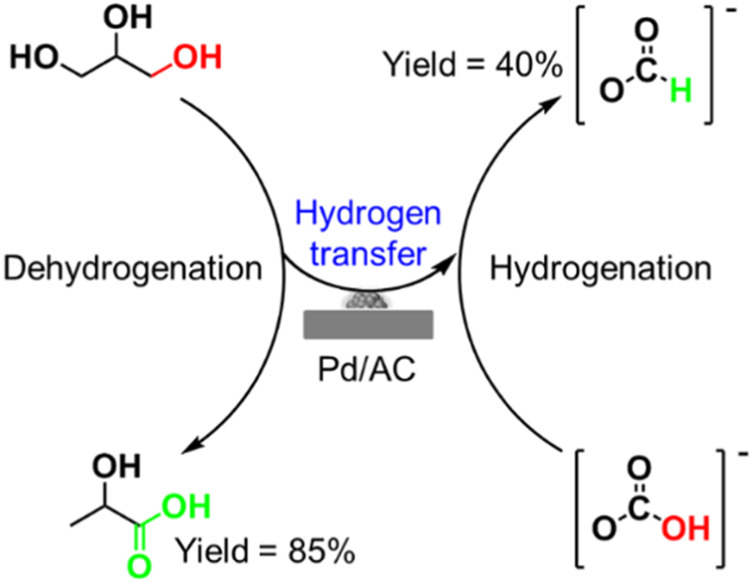
Dehydrogenation and catalytic transfer hydrogenation of glycerol and CO_2_/HCO_3_^−^.

Su et al. (2014) reported solid Pd/AC (AC: activated carbon) catalyzed one-pot tandem dehydrogenation and catalytic transfer hydrogenation of glycerol and carbonate/bicarbonate to value-added carboxylic acids (Scheme 5). High yield of LA (55%) and FA (29%) were achieved in 12 h at 240°C under 400 psi N_2_ pressure. A general controversy about the one-pot hydrogen transfer reaction is the pathway for the formation of FA. They carried out a series of control reactions in the absence of glycerol or HCO_3_^−^. No FA was observed in reaction products, indicating that FA is formed by the hydrogenation of HCO_3_^−^ instead of the degradation of glycerol. Notably, in their work, both CO_3_^2-^ and HCO_3_^−^ were much easier to be hydrogenated than CO_2_ gas, which is different from electrochemical reduction of CO_2_. The highest FA yield reached 42%, while using CO_3_^2-^ as an H_2_ acceptor. However, only few FA (yield of 1.2%) and 22 turnovers were actually obtained in 12 h at 240°C, while directly using CO_2_ as an H_2_ acceptor. They also studied the possible hydrogen transfer routes in detail. Combined XRD and XPS analysis with experimental data, they proposed the plausible direct hydrogen transfer mechanism for the one-pot tandem dehydrogenation and catalytic transfer hydrogenation between glycerol and CO_2_ to LA as well as FA. The aforementioned tandem reaction would be strongly limited by the active sites of the Pd nanoparticles, because the co-adsorption of glycerol and HCO_3_^−^ could be rate limiting. Their work proved that the one-pot catalytic transfer hydrogenation is feasible combined with the dehydrogenation of glycerol and hydrogenation of carbonate/bicarbonate. High yield of LA (∼85%) and FA (∼40%) as a value-added hydrogenation product was finally obtained under certain reaction conditions.

## Tandem dehydrogenation and catalytic transfer hydrogenation reaction of glycerol

**FIGURE 5:**
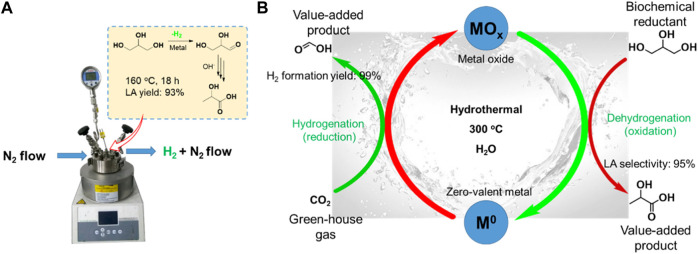
**(A)** Conversion of glycerol to LA under N_2_ flow. **(B)** Carbon cycles with biochemical as reductants via M^0^/MO_x_ redox cycles (Jin et al., 2011).

One-pot dehydrogenation and catalytic transfer hydrogenation of glycerol with H_2_ acceptor is a greatly complex parallel reaction, needing a good balance in dehydrogenation and hydrogenation reaction in a synchronized time. Thus, it is difficult to obtain a high yield of LA and FA at the same time (Heltzel et al., 2018). Two-pot reaction, separating dehydrogenation and hydrogenation processes, maybe a good strategy for efficient recovery of valuable hydrogen while achieving a high yield of LA. Recently, Siddiki et al. (2017) reported that the LA yield would be significantly enhanced via rapid removal of the released H_2_ from dehydrogenation of glycerol (Figure 5A). They compared the conversion of glycerol with O_2_ flow and static O_2_ pressure as well as N_2_ flow and static N_2_ pressure under the same reaction conditions (0.03 mol% Pt/AC for glycerol, 1.1 equiv. KOH, 160°C, 18 h). Under O_2_ or N_2_ flow conditions, the Pt/AC catalyzed reaction gave a significantly enhanced yield of LA (75% and 93%, respectively), but greatly reduced the hydrogenation yield (6% and less than 2%, respectively). However, under static O_2_ or N_2_ pressure in a closed reactor, the LA yield was only 56% and 59%, respectively, while the yield of hydrogenation products including 1,2-PDO, EG, and other alcohols reached up to 20%. These results indicate that the rapid removal of the released H_2_ from dehydrogenation of glycerol could obviously suppress the undesirable hydrogenation reaction for conversion of glycerol to LA. Compared with oxidation of H_2_ by O_2_, it is clear that purging the H_2_ by flowing N_2_ before it goes into the hydrogenation reaction is more effective for producing LA. Furthermore, the released H_2_ can be collected and used in many fields, including the ammonia synthesis, petrochemical, Fishcher–Tropsch process, and clean energy industry. Even more, we can design the two-pot catalytic transfer hydrogenation system by connecting partial dehydrogenation of glycerol and hydrodeoxygenation reaction with various biomass-derived substrates.

In a typical case, glycerol can be converted into value-added chemicals via aqueous-phase hydrodeoxygenation (APH) reaction (Jin et al., 2019). Hence, we can design a two-pot tandem dehydrogenation and APH reaction for converting glycerol to achieve both good activity and selectivity for LA and 1,2-PDO. Nevertheless, a recycling system is still needed to separate released H_2_ from dehydrogenation of glycerol, which demands further consideration for industrial applications. Furthermore, APH of glycerol requires relatively high H_2_ pressure and temperature to increase the hydrogenation rate, leading to undesirable methanation reaction (Roy et al., 2010). From a molecular point of view, the difficult dissolution of molecular H_2_ would also reduce the intrinsic kinetics of hydrogenation reactions (Jin et al., 2019). Therefore, it is clear that there is a strong impetus to improve overall atomic and energy efficiency of tandem dehydrogenation and catalytic transfer hydrogenation technologies for achieving both high LA yield and valuable utilization of H_2_ released from dehydrogenation of glycerol.

**SCHEME 6:**
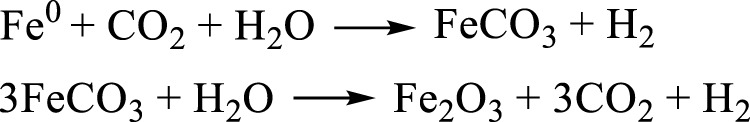
Possible mechanism for hydrogen generation with Fe^0^ (Jin et al., 2011).

Jin et al. (2011) (Yao et al., 2017) disclosed a strategy for achieving both dehydrogenation of glycerol to LA and reduction of CO_2_ to FA via a two-pot tandem redox reaction catalyzed by the transition metal (Figure 5B). A cycle can be achieved using the oxidative potential of zero-valent metals to reduce CO_2_ to FA in the presence of water and the reductive potential of glycerol to reduce the metal oxides to their zero-valent state. As an oxidation product, LA is produced in reduction of MO_x_ to M^0^. Furthermore, the H_2_ for hydrogenation of CO_2_ is formed from water. For example, Fe metal first reacts with CO_2_ and H_2_O to release H_2_, following the resulting FeCO_3_ which undergoes hydrolysis to form Fe_2_O_3_ and another molecule of H_2_ (Scheme 6). Metals including Zn, Al, and Mn have been demonstrated to be feasible for producing H_2_ under similar mechanism, where they reported that a maximum H_2_ yield of 99% was achieved. The MO_x_ could be reduced by glycerol to M^0^, and LA with a high selectivity of 95% was produced at the same time. The principle of the tandem redox reaction in CO_2_, glycerol and metal/metal oxide is schemed in Figure 5B. In their work, the dehydrogenation of glycerol to LA and the hydrogenation of CO_2_ to FA are connected by the redox reaction of a series of metal/metal oxide. The valuable utilization of H_2_ released from glycerol dehydrogenation is also achieved by a medium of metal–metal oxide pairs.

## Conclusion and outlook

Due to the intense interest in the reaction pathways of atomic economy during process development, experimental, and theoretical studies on combined dehydrogenation of glycerol to LA and catalytic transfer hydrogenation of H_2_ acceptors to chemicals are receiving increased interest. In this review, plausible reaction pathways and mechanisms for catalytic upgradation of glycerol into LA under both aerobic and anaerobic conditions, one-pot/tandem dehydrogenation and catalytic transfer hydrogenation between glycerol and H_2_ acceptors have been critically reviewed with the aim to provide insights into future development of the reaction pathways of atomic economy during process development in catalytic upgradation of unconventional resources to value-added fuels and chemicals. A variety of different H_2_ acceptors have been proposed with remarkable performance for transfer hydrogenation with released H_2_ from dehydrogenation of glycerol. Plausible reaction pathways and mechanisms have been well documented in the current work.

However, two challenges still need to be resolved for catalytic conversion of glycerol to LA with atomic economic reaction pathways:

1) Matching the reaction rates of H_2_ release and consumption during dehydrogenation of glycerol to LA and catalytic transfer hydrogenation of H_2_ acceptors. One-pot dehydrogenation and catalytic transfer hydrogenation of glycerol with H_2_ acceptor is a greatly complex parallel reaction, needing a good balance in dehydrogenation and hydrogenation reactions in a synchronized time. However, there is still demand for a dual-function catalyst with more activity of catalytic transfer hydrogenation of H_2_ acceptors to match the reaction rates of H_2_ release and consumption. It is expected that the novel catalyst can simultaneously improve the yield of LA and hydrogenation products.
2) Main stream research efforts have still been focused on enhancement of the yield of catalytic conversion of glycerol to LA, rather than the yield of hydrogenation products. So far, various H_2_ acceptors, especially cyclohexene, 1-decene, levulinic acid, nitrobenzene, benzene, and ethylene gas, significantly enhance the formation of LA during catalytic conversion of glycerol. However, these H_2_ acceptors are transformed into undesirable cheaper chemicals. Using CO_2_ and its derivatives as H_2_ acceptors is a good solution, because the hydrogenation products of these H_2_ acceptors are general value-added chemicals. In addition, catalytic transformation of CO_2_ to value-added chemicals or fuels provides the possibility for the carbon neutrality and sustainable development of human society. To improve hydrogenation activity and yield, it is necessary to understand H species generation from glycerol, transfer and hydrogenation with H_2_ acceptors. Moreover, the rational design of dual-functional (dehydrogenation and hydrogenation) catalysts still demands further experimental efforts in future studies.
